# PF74 and Its Novel Derivatives Stabilize Hexameric Lattice of HIV-1 Mature-Like Particles

**DOI:** 10.3390/molecules25081895

**Published:** 2020-04-20

**Authors:** Alžběta Dostálková, Kryštof Škach, Filip Kaufman, Ivana Křížová, Romana Hadravová, Martin Flegel, Tomáš Ruml, Richard Hrabal, Michaela Rumlová

**Affiliations:** 1 Department of Biotechnology, University of Chemistry and Technology, 166 28 Prague, Czech Republic; dostalkl@vscht.cz (A.D.); kaufmanf@vscht.cz (F.K.); krizovaa@vscht.cz (I.K.); romana.hadravova@uochb.cas.cz (R.H.); 2 Department of Chemistry of Natural Compounds, University of Chemistry and Technology, 166 28 Prague, Czech Republic; K.Skach@seznam.cz (K.Š.); martin.leglef@gmail.com (M.F.); 3 Department of Biochemistry and Microbiology, University of Chemistry and Technology, 166 28 Prague, Czech Republic; tomas.ruml@vscht.cz; 4 NMR Laboratory, University of Chemistry and Technology, 166 28 Prague, Czech Republic; hrabalr@vscht.cz

**Keywords:** HIV-1 CA inhibitor, PF74 derivatives, uncoating, disassembly

## Abstract

A major structural retroviral protein, capsid protein (CA), is able to oligomerize into two different hexameric lattices, which makes this protein a key component for both the early and late stages of HIV-1 replication. During the late stage, the CA protein, as part of the Gag polyprotein precursor, facilitates protein–protein interactions that lead to the assembly of immature particles. Following protease activation and Gag polyprotein processing, CA also drives the assembly of the mature viral core. In the early stage of infection, the role of the CA protein is distinct. It controls the disassembly of the mature CA hexameric lattice i.e., uncoating, which is critical for the reverse transcription of the single-stranded RNA genome into double stranded DNA. These properties make CA a very attractive target for small molecule functioning as inhibitors of HIV-1 particle assembly and/or disassembly. Of these, inhibitors containing the PF74 scaffold have been extensively studied. In this study, we reported a series of modifications of the PF74 molecule and its characterization through a combination of biochemical and structural approaches. Our data supported the hypothesis that PF74 stabilizes the mature HIV-1 CA hexameric lattice. We identified derivatives with a higher in vitro stabilization activity in comparison to the original PF74 molecule.

## 1. Introduction

Capsid protein (CA) is the main structural protein of retroviral particles. CA consists of two helical domains, the N-terminal (CA-NTD) and C-terminal (CA-CTD) domains, which are connected by a short linker. As a part of structural polyprotein Gag, CA mediates intra- as well as inter-molecular interactions that lead to the formation of a hexameric lattice of immature particle. Later on, following retroviral protease activation, the Gag polyprotein is processed to individual structural proteins and released as CA molecules re-assembled into mature hexameric lattice that form a viral core or capsid. Despite a low amino acid sequence similarity, both CA domains share similar secondary and tertiary structures among various retroviral genera. However, the quaternary structures of CA domains within the immature lattices of various retroviruses, such as Mason-Pfizer monkey virus, Human immunodeficiency virus 1 (HIV-1), Rous sarcoma virus, and Murine leukemia virus, are different [1,2,3,4]. In contrast to a purely hexameric, immature lattice, which can be bended and closed only due to several openings (gaps) in the structure [5], the properly assembled mature hexameric lattice can form fully closed fullerene or multilayered structures [1]. This is enabled by the accommodation of twelve pentamers in the hexameric structure of mature retroviral cores [1,6,7,8,9]. Researchers have proposed that a variety of retroviral core architectures (e.g., conical, tubular, or pleiomorphic) are determined by the position of pentamers [10,11,12]. 

In addition to the key role of CA during the assembly of both immature and mature types of retroviral particles, CA is also critical during virus uncoating. Uncoating is a poorly understood post-entry event in the early stage of the retroviral life cycle, comprising controlled disassembly of the core upon entering the cytoplasm. Even though this process has been intensively studied, the exact subcellular location, mechanism, and timing of retroviral uncoatings are unclear and the data are controversial [13,14,15,16,17,18,19,20,21]. Based on several studies showing that the host cell proteins bind to the assembled CA lattice it is plausible that the viral core persists largely intact upon entry to the cytoplasm [22,23]. However, whether the uncoating is accomplished in the cytoplasm or at the nuclear pore remains unclear [13,15,16,18,19,20,24,25]. A recent study demonstrated that multiple CA proteins can direct the viral genome into the nucleus of the infected cell [26]. HIV-1 capsid disassembly is tightly connected to reverse transcription as its inhibition delays the uncoating [19,27,28]. HIV-1 uncoating is also linked to binding of a variety of cellular proteins and small cofactor molecules [29,30,31,32,33,34,35,36,37,38]. Due to the above mentioned importance of CA in various steps of the HIV-1 replication cycle, mutations in CA are very often fatal for the virus [13,39,40]. Therefore, researchers expected that the mutations within CA developed against CA-targeting drugs might compromise the fitness of the virus [39]. 

The importance of CA in many essential functions, such as the assembly of immature particles, the formation of the mature core, and the controlled disassembly, makes it a highly attractive pharmacological target [41]. Indeed, studies reported several capsid-binding small molecules that inhibited HIV-1 replication, for review see [42,43,44]. Recent studies suggested that these drugs blocking viral nuclear import by targeting the viral CA could be a successful therapeutic approach [45]. Based on all the above mentioned recent knowledge, the development of new compounds that can directly and specifically block the early steps of viral infection, and probably also CA assembly, are highly in demand.

**Figure 1:**
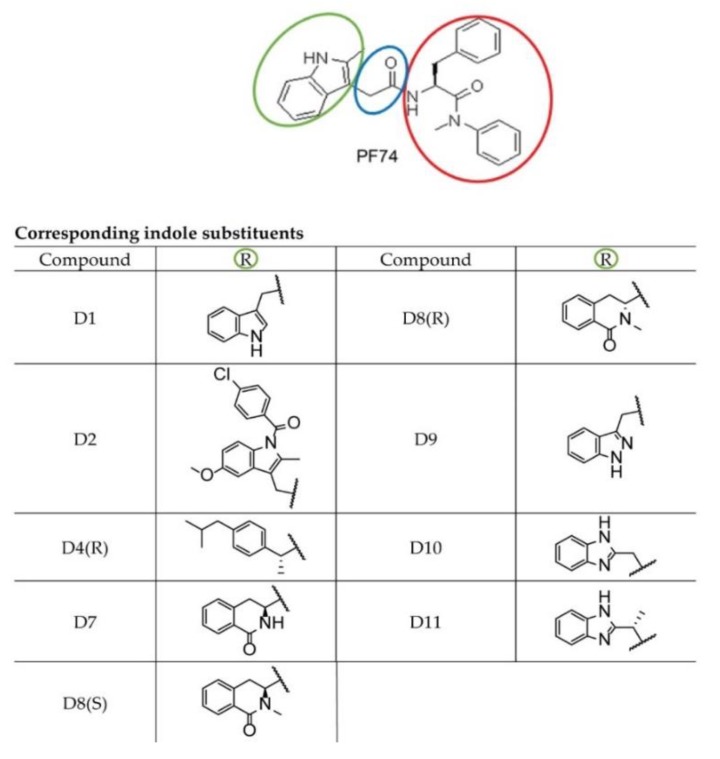
PF74 and its derivatives. Chemical structures of PF74 and its indole derivatives used in this study.

The PF-3450074 (PF74) molecule (Figure 1), binding to CA lattice is one of the most studied and most promising inhibitors of HIV-1 [45,46]. The PF74 molecule, first identified by Pfizer, USA [47], was reported to inhibit HIV-1 replication [48,49,50]. PF74 binds to the HIV-1 CA hexameric lattice and affects the stability of mature capsid. However, whether PF74 stabilizes or destabilizes the hexameric lattice is still unclear [47,49,51,52,53]. The structure of PF74 consists of two components: a phenylalanine part (Figure 1, red) and an indole substituent (Figure 1, green), which are connected by a linker (Figure 1, blue). PF74 binds by its phenylalanine core to the pocket within the CA-NTD formed by the helices H3, H4, H5, and H7, while the indole ring interacts with the CA-CTD of the adjacent subunit [22,47,51,54].

To improve the poor therapeutic qualities and poor metabolic stability [55], several efforts to modify PF74 were reported [45,46,55,56]. In this work, we report several modifications of the indole and linker part of the PF74 molecule. To quantify the effect of PF74 derivatives on the stability of the viral capsid, we used a combination of in vitro, cell-based, and NMR structural assays. The data gained by our recently developed in vitro disassembly inhibitor test for HIV (DITH) [57] strongly supported the hypothesis that the mechanism of PF74 is to stabilize, not to destabilize, the mature HIV-1 CA hexameric lattice. Moreover, the higher in vitro stabilization activity of the D9 and D10 derivatives in comparison to the original PF74 pointed toward the nature of further possible modifications.

## 2. Results and Discussion 

### 2.1. The Design and Synthesis of PF74 Derivatives

To enhance the inhibiting capability of PF74, we designed and synthesized a series of modified PF74 derivatives comprising the indole substituent and connected linker (Figure 1: green and blue, respectively). The capability to interact with the PF74 binding sites of CA was tested in silico by the docking program Glide Schrödinger, USA). In all cases, the HIV-1 CA N-terminal binding part (Figure 1: red) remained intact and the modifications targeted only the C-terminal binding indole part (Figure 1: green) and the linker (extended by one carbon in D11). 

### 2.2. Analysis of PF74 Derivatives Activity Using in Vitro Protein-Based Methods

#### 2.2.1. Fast Assembly Inhibitor Test for HIV (FAITH) Analysis

**Figure 2:**
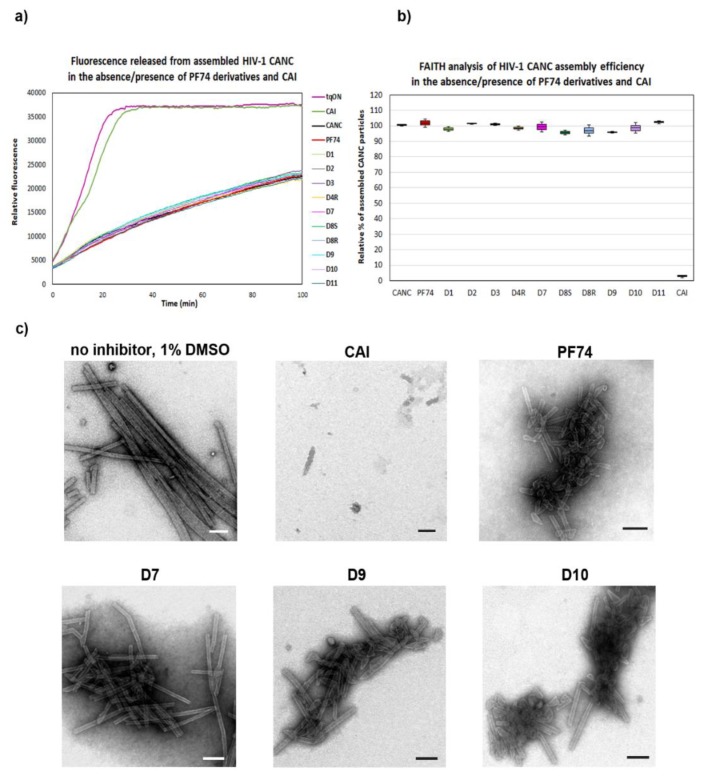
Quantification of the in vitro assembly of HIV-1 CANC in the presence of PF74 and its derivatives by Fast Assembly Inhibitor Test for HIV (FAITH). HIV-1 CANC (18 µM) was mixed with tqON (1.8 µM) CAI, and PF74 or its derivatives to a final concentration 10 µM. Following the assembly reaction, Exonuclease I was added to each sample to degrade free, non-incorporated tqON. (**a**) Fluorescence released from degraded tqON was monitored for tqON itself (purple), CANC without inhibitor (black) and from the sample containing. DMSO without inhibitor (black) and from the sample containing CAI (green), PF74 (red), D1 (light green), D2 (grey), D4R (yellow), D7 (pink), D8S (dark green), D8 (light blue), D9 (cyan), D10 (violet), and D11 (azure). (**b**) Assembly efficacy was calculated from the difference in relative fluorescence between the control represented by free tqON (panel a, purple curve) and the assembled CANC particles in the absence of an inhibitor (panel a, black curve) or in presence of inhibitors. The relative percentage of the efficacy of HIV-1 CANC assembly in the presence of PF74 and its derivatives was compared to that of CANC, which was considered as 100%. (**c**) Following FAITH, in vitro assembled HIV-1 CANC in the presence of indicated inhibitors were negatively stained and analyzed by TEM. Bar represents 200 nm.

To analyze the effects of the PF74 derivatives on the HIV-1 core assembly, disassembly, and stability in vitro, we first employed the Fast Assembly Inhibitor Test for HIV (FAITH) [58]. Using recombinant HIV-1 capsid-nucleocapsid (CANC) protein and dually labeled TaqMan-like oligonucleotide (tqON), this assay provides quantitative information regarding the efficiency of the assembly of HIV-1 mature-like particles. During the three-hour assembly reaction, HIV-1 CANC and tqON formed mature-like structures. Inside these assemblies were hidden the tqON molecules, which were bound by the NC domains and packaged into the forming particles. When Exonuclease I was added to the reaction mixture, it degraded those tqON that were not packaged inside the particles and remained free in the solution. During tqON degradation, a reporter dye-fluorescein (FAM) was separated from its quencher molecule-black hole quencher (BHQ), and the FAM fluorescence emission was measured (Figure 2a, purple curve). 

As the fluorescence is released from the non-incorporated, free tqONs but not from those packaged and protected by the CANC assembled particles, the amount is proportional to the efficiency of the CANC assembly. To analyze the effects of the PF74 derivatives on the assembly of mature-like HIV-1 particles, we mixed the tested inhibitors with HIV-1 CANC protein and, following a one hour incubation, the assembly reaction was initiated by tqON addition. Next, Exonuclease I was added, and the fluorescence release, due to the degradation of free, non-incorporated tqONs, was measured (Figure 2a). The level of assembly efficiency was then calculated from three independent measurements as a difference in the relative fluorescence between the control represented by free tqONs (Figure 2a, purple curve) and the assembled CANC particles in dimethyl sulfoxide (DMSO) in the absence of inhibitor (CANC, black curve) or in presence of inhibitors (Figure 2a). The relative percentage of the efficacy of the HIV-1 CANC assembly in the presence of PF74 and its derivatives was then compared to that of the CANC without inhibitor, which was considered as 100% (Figure 2b).

As expected, in contrast to the control peptide inhibitor CAI [59] that completely abolished the CANC assembly (Figure 2), no effect of PF74 and its derivatives on the assembly of mature-like CANC particles was observed by FAITH (Figure 2a,b). In accord with that, transmission electron microscopic analysis of selected negatively stained assembled samples showed the presence of typical tubular and cone-like structures in the tested samples (Figure 2c), confirming the observation that none of the PF74 derivatives affected the in vitro assembly of HIV-1 mature particles. In the absence of an inhibitor, CANC typically assembled into long, tubular structures. The same phenotype was observed for CANC assembled in the presence of the D7 derivative. However, the predominant formation of cone-like and shorter tubular structures was observed for HIV-1 CANC particles assembled in the presence of PF74 and D10 inhibitors (Figure 2c). 

#### 2.2.2. DITH Analysis

**Figure 3:**
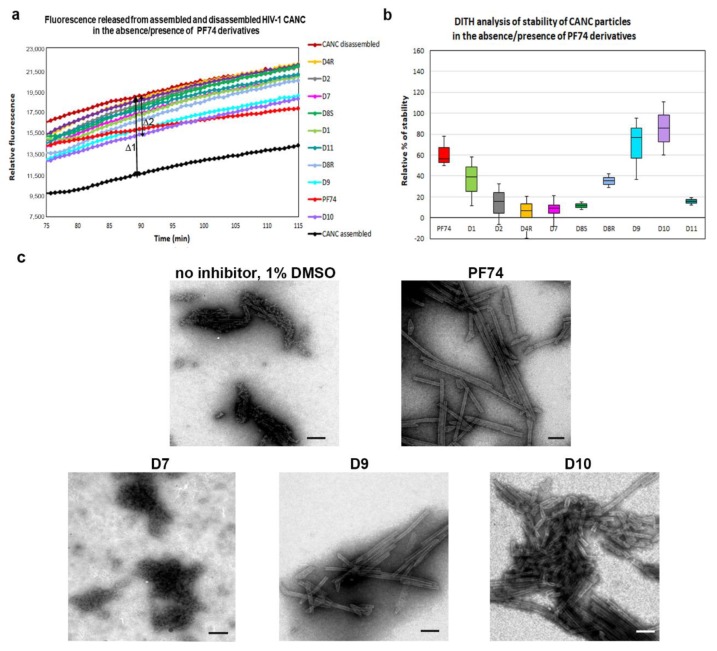
Disassembly inhibitor test for HIV (DITH) analysis of the effect of PF74 and its derivatives on the stability of HIV-1 CANC. (**a**) Graph showing the fluorescence emission curves demonstrating the kinetics of tqON release from preassembled CANC particles incubated in assembly buffer (black curve) and disassembly buffer (red curve) in the absence of PF74 derivatives or in the disassembly buffer containing: PF74 red, D1 light green, D2 grey, D4R yellow, D7 pink, D8S dark green, D8R light blue, D9 cyan, D10 violet, and D11 azure at the final concentration 10 µM. The stabilization effect of PF74 and its derivatives was calculated as the difference between the relative fluorescence of tqON at 90 min in the disassembly and assembly reactions according to the calculation: relative percent of stabilization = 100*Δ2/Δ1. (**b**) DITH quantification of the relative stability of preassembled CANC particles incubated in the disassembly buffer in the presence of PF74 and its derivatives, measured and calculated as described in (**a**). The relative stability of CANC in the absence of inhibitor in disassembly buffer was considered as 0%. (**c**) Following DITH, the in vitro assembled HIV-1 CANC in the presence of indicated inhibitors were incubated in disassembly buffer, negatively stained, and analyzed by TEM. Bar represents 200 nm.

In contrast to the assembly of immature particles, PF74 was reported to affect the stability of the hexameric lattice of HIV-1 mature particles during disassembly. To quantify and compare the effects of PF74 and its derivatives on the stabilization/destabilization of the CA hexameric lattice of CANC tubular structures, we next applied the DITH assay [57]. Similarly to FAITH, this method was based on the measurement of fluorescence released from the tqON. DITH uses preassembled CANC-tqON complexes that are incubated under disassembly conditions in the presence or absence of PF74 and its derivatives. The amount of fluorescence signal released from tqON is then proportional to the level of HIV-1 CANC disassembly. The stabilization or destabilization effect of tested compounds can be then calculated. We assembled CANC tubes in assembly buffer in the presence of tqON. Following 1 h incubation of the CANC tubes with DMSO (control sample) or PF74 and its derivatives (final concentration 10 µM), the particles were diluted into disassembly buffer and incubated overnight at laboratory temperature. Immediately following the Exonuclease I addition, the fluorescence was measured (Figure 3a) and the relative percentage of CANC stability was calculated for each sample (Figure 3b).

The relative percentage of stability of HIV-1 CANC mature particles in the presence of PF74 and its derivatives was determined as the difference between fluorescence of degraded tqON at 90 min in disassembly and assembly reactions for the CANC and PF74 derivatives treated CANC assemblies (Figure 3a, Δ1 and Δ2, respectively). The relative percentage of PF74 derivatives-mediated stabilization was determined using the formula: 100* Δ2/Δ1 and compared to that of the wild type in the disassembly buffer whose stability was considered as 0% (Figure 3b). The stability of PF74-treated HIV-1 CANC assemblies in disassembly buffer was about 60% higher compared to the control CANC sample. Compounds D9 and D10 turned out to be more potent stabilizers than PF74, increasing the CANC assembly stability to 80% and 90%, respectively. 

The D1 compound was only slightly less active in stabilization than PF74. All other tested PF74 derivatives (D2, D4R, D7, D8R, D8S, and D11) showed a lower effect on HIV-1 CANC stability than PF74 (Figure 3b). TEM analysis of the samples following DITH (Figure 3c) confirmed the presence of the intact CANC particles in the samples containing PF74 or compounds that increased the CANC particle stability in the DITH assay (D9 and D10). In contrast, TEM analysis of the CANC particles disassembled in the absence of PF74 or in the presence of the non-active inhibitors (D2, D4R, D7, D8R, D8S, and D11) showed mainly disassembled material. These data clearly support the observation that a PF74 inhibitor stabilized the mature hexameric lattice and thus inhibited the processes connected to uncoating. A similar conclusion was reported based on the measurement of HIV-1 CA cores stiffness, which was enhanced in the presence of PF74 [52]. 

### 2.3. Analysis of PF74 Derivatives Using Cell-Based Methods

#### 2.3.1. Effect of PF74 Derivatives on HIV-1 Infectivity

First, using a resazurin assay, we tested the cytotoxicity of PF74 derivatives (Table 1). None of the tested inhibitors were cytotoxic, with the exception of D10 with cytotoxic concentration CC_50_ value 10 µM and D4R CC_50_ 27 µM. Then we verified the impact of PF74 derivatives on HIV-1 infectivity using a single-round HIV infectivity assay. HIV-1 particles pseudotyped with vesicular stomatitis virus (VSV) glycoproteins that were produced in human embryonic kidney (HEK 293) cells. At 48 h post-transfection, the content of HIV-1 CA protein in the culture media was quantified by ELISA, and normalized amounts of VSV-G pseudotyped HIV-1 containing green fluorescent protein (GFP) were used to infect fresh HEK 293 cells. Immediately after infection, various concentrations of PF74 or its derivatives were added to the cells (DMSO was used for the control). At 48 h post-infection, the HIV-1 infectivity was determined by the quantification of GFP-positive cells using flow cytometry, and the 50% inhibition concentration (IC_50_) value for each inhibitor was calculated. The IC_50_ was defined as the concentration of compound that reduced the HIV-1 infectivity by 50% compared to the DMSO-treated control. The D10 derivative revealed a better inhibitory activity against HIV-1 than PF74. The activity of three other derivatives D1, D8R, and D9 was only slightly weaker than that of PF74. These data nicely correlated with an in vitro stabilization assay (Figure 3b).

#### 2.3.2. Cyclosporin A (CsA)-Washout Assay 

The DITH results suggested that three of the PF74 derivatives (D1, D9, and D10) stabilized the HIV-1 CA lattice in vitro more efficiently than the PF74 molecule. To verify these in vitro data and evaluate the DITH assay in comparison with a cell-based method, we also tested the inhibitors using the CsA-washout assay. This assay monitored the HIV-1 capsid core uncoating (or disassembly) within infected cells [19,60]. The CsA-washout assay is based on the fact that the endogenously expressed—e.g., in owl monkey kidney (OMK)—HIV-1 restriction factor, tripartite motif-containing protein 5 (TRIM5α)-Cyclophilin A (CypA), specifically binds to the CA hexameric lattice of the mature HIV-1 core and blocks viral infection. However, in the presence of CsA, HIV-1 loses sensitivity to its restriction factor. In the CsA-washout assay, CsA is gradually washed out of the cells, thus enabling TRIM5α-CypA binding to the CA lattice and blocking HIV-1 infection [19]. To test the PF74 derivatives in this assay, we first tested the cytotoxic effect of the PF74 compounds on OMK cells. Unfortunately, the promising D10 derivative showed a CC_50_ below 3 µM which prevented its further testing in the CsA-washout analysis. To test the other PF74 derivatives, the OMK cells were transfected with an ELISA-normalized amount of VSV-G pseudotyped GFP-HIV-1 virions in the presence of CsA and inhibitors or DMSO (as a control). 

**Figure 4:**
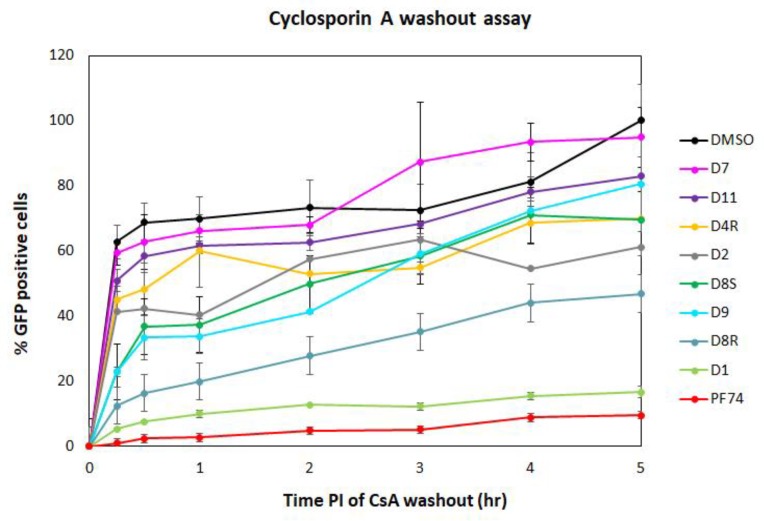
CsA-washout assay. Owl monkey kidney (OMK) cells were infected by spinoculation with an ELISA-normalized amount of VSV-G pseudotyped HIV-1 in the presence of CsA and DMSO, PF74 and its derivatives. Inhibitors were added to the HIV-1 sample before spinoculation at a final concentration of 10 µM. At the indicated times post-infection, the CsA-containing medium was replaced with fresh, CsA-free culture medium. The percentage of GFP-positive OMK cells was determined by flow cytometry using a BD FACS Aria III (Becton Dickinson) flow cytometer and the data were analyzed with Diva 8 software. The number of GFP-positive cells measured at the indicated time was then calculated and normalized to the DMSO-containing non-drug control reaction, which was considered as 100%.

Following the CsA-washout at various time intervals, the percentages of GFP-positive (i.e., infected) cells were determined by flow cytometry, normalized to the non-drug control reaction (in DMSO), by setting the percentage at 5 h to 100%, and used in the graph (Figure 4). PF74 and D1 derivative showed the strongest impact on HIV-1 infectivity in OMK cells at the early stage of HIV-1 infection. All other PF74 derivatives appeared to affect the uncoating, and D8R had a major effect. The D9 derivative appeared to be highly active during the first two hours post infection, then its activity dropped. 

### 2.4. NMR Analysis of the Binding Mode of D10

**Figure 5:**
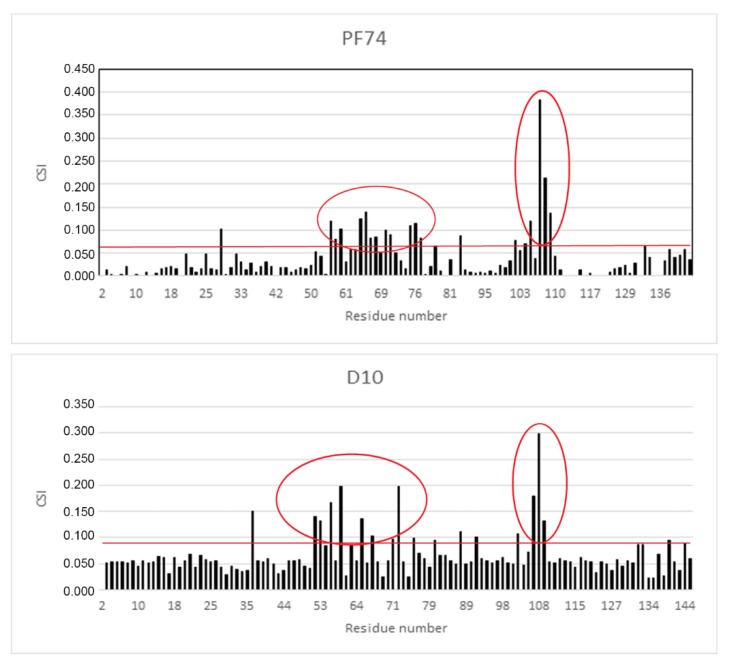
The chemical shift index of binding of PF74 and D10 to CA-NTD. Chemical shift changes, caused by DMSO, were subtracted from the experimental changes caused by the addition of inhibitors dissolved in DMSO. The horizontal red lines indicate the cut off, which was calculated as the average chemical shift index values (CSI) of all amino acids plus one standard deviation. The red ovals highlight the areas of substantial CSIs corresponding to the amino acids of the binding site.

As the derivative D10 was the most active stabilizer in in vitro analysis, we determined its binding mode by measuring a series of 2D ^1^H-^15^N heteronuclear single quantum correlation (HSQC) experiments on ^15^N-labeled CA-NTD with increasing concentration of the inhibitor and comparing the results with the data for PF74. Figure 5 shows the histograms of chemical shift index values (CSI) for all amino acid residues of CA-NTD with bound PF74 and D10. 

CSIs were calculated from the experimental data according to the formula

(1) $$d=\sqrt{\frac{1}{2}\left[\delta_{H}^{2}+\left(\alpha \times \delta_{N}^{2}\right)\right]}$$

where δ_H_ and δ_N_ are ^1^H and ^15^N; respectively, chemical shift changes and α is a weighting factor (0.2 in this case).

**Figure 6:**
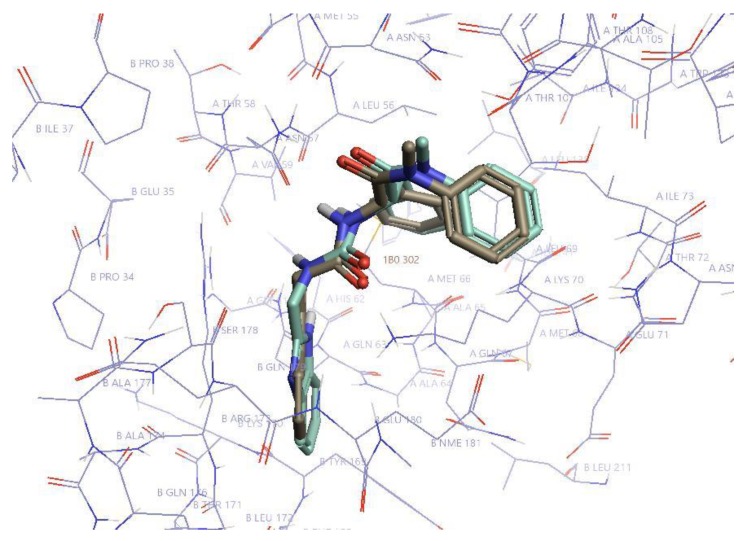
The structures of PF74 and D10. PF74 is in khaki and D10 in pale green. Hydrogen atoms are omitted for simplification. The most important residues of CA-NTD (**A**) and CA-CTD (**B**) for the inhibitor binding are labeled. Created in Flare (Cresset Inc., New Cambridge House, Bassingbourne Road, Littlington, Cambridgeshire SG8 0SS, UK).

The most affected amino acid residues in both molecules were Thr 54, Leu56, Asn57, Gln63, Ala64, Met66, Gln67, Leu69, Lys70, Asn74, Glu75, Glu76, Asp103, Ile104, Gly106, Thr107, and Thr108. This is in accordance with the binding site of PF74 to CA-NTD, as it was previously determined by X-ray crystallography [51]. In silico docking of D10 resulted in the same binding mode, which was confirmed by highly similar histograms. The comparison of the structures of D10 and PF74 is shown in Figure 6.

The published data concerning the effects of PF74 are rather contradictory. Some experiments proved that PF74 decreased the stability of the HIV-1 core and thus accelerated its disassembly [49]. However, another paper documented that PF74 strengthened the stability of the HIV-1 CA cores, and thus slowed down the disassembly process [51,52,57]. Our data, obtained using an in vitro stabilization assay (DITH) supported the model in which PF74 acted as a stabilizer of the HIV-1 mature hexameric lattice [51]. Two of the PF74 derivatives with a modified indole moiety, D9 and D10, revealed higher in vitro stabilization activity when compared to PF74. In order to diminish the D10 cytotoxicity, further modifications are currently under our investigation. 

## 3. Materials and Methods 

### 3.1. Expression Vector Preparation

HIV-1 CANC, encoding CA, SP1, and the NC fusion protein was prepared as described earlier [58,61]. Three vectors: the packaging psPAX2, encoding HIV-1 Gag, Pol, Tat, and Rev; the reporter/transfer pWPXLd-GFP, encoding long terminal repeat (LTR), rev-response element (RRE), and GFP and envelope pHEF-VSV-G, encoding vesicular stomatitis virus Env (VSV-G) were used for the production of pseudotyped VSV-G HIV-1 particles. The psPAX2 vector [62] was kindly provided by Dr. Jeremy Luban, UMASS Medical School, Worcester, MA, USA) and the pWPXLd-GFP and pHEF-VSV-G vectors were purchased from Addgene (Cambridge, MA, USA).

### 3.2. Expression and Purification of HIV-1 CA-Derived Proteins

The HIV-1 CANC protein was purified as previously described [58,61,63]. Briefly, the HIV-1 CANC protein was expressed in *Escherichia coli (E.coli)* BL21 (DE3) and following cell lysis, polyethyleneimine to a final concentration of 0.15% (*w*/*v*) was added to the cell lysate and the nucleic acids were removed by ultracentrifugation (Beckman (Brea, CA, USA), TI 90, 55,000 rpm, 3 h, 4 °C). The HIV-1 CANC protein was purified by a combination of ion-exchange chromatography using HiPrep™SP FF 16/10 column (GE Healthcare, Chicago, IL, USA) and gel-filtration chromatography using HiLoad™26/600 Superdex™ column. The HIV-1 CANC protein was concentrated to 4 mg/mL and stored at −80 °C. 

### 3.3. Fast Assembly Inhibitor Test for HIV (FAITH)

The assay was used to quantify the effect of PF74 derivatives on the assembly of mature-like HIV-1 particles, as described in [58]. In 96-well plate, we pre-incubated the HIV-1 CANC protein (60 µg/100 µL) with the tested inhibitor (final concentration 10 µM) and kept it on ice for 1 h. To start the assembly reaction, 3 µg of dually labelled oligonucleotide (tqON) was added to the CANC protein and the volume of the reaction mixture was adjusted to 100 µL using the assembly buffer (50 mM Tris, pH 8.0, 1 µM ZnCl_2_, 340 mM NaCl). Following a 3-h incubation at room temperature, Exonuclease I (ExoI) 6 mM Mg^2+^ was added and the fluorescence of the fluorophore released from degraded tqON was measured using a Tecan M200Pro plate reader.

### 3.4. Stabilization Fast Assembly Inhibitor Test for HIV (DITH)

This assay was used as described in [57]. Tested inhibitors were added at a final concentration of 10 µM to HIV-1 CANC tubular structures in a 96-well plate, assembled as described for FAITH. The mixture of CANC protein and tqON was incubated for 3 h at room temperature. Then, inhibitors were added and incubation continued for 1 h. Next, we added 100 µL of disassembly buffer (50 mM Tris, pH 7.0, 1 µM ZnCl_2_) to the each sample in the plate, and incubation, under moderate agitation, continued overnight at room temperature. ExoI and Mg^2+^ ions were added 16 h later, and the fluorescence was measured as for FAITH. 

### 3.5. Determination of Cytotoxicity Using Resazurin Assay

The human embryonic kidney cells (HEK 293T) were seeded in a 48 well plate (3 × 10^5^cells/mL) in DMEM medium supplemented with 10% FBS in 5% CO_2_ atmosphere at 37 °C. The next day, the cells were treated with various concentrations of PF74 and its derivatives, ranging from 10–50 µM. We added resazurin of a final concentration of 25 µg/mL, 24 h later, to each sample. The cells were incubated for an additional 4 h, in 5% CO_2_ atmosphere at 37 °C in the dark. The metabolic activity of the cells was then measured using a microplate reader (Tecan M200Pro) at 560 nm excitation/590 nm emission wavelength. All experiments were done in triplicates.

### 3.6. Production of VSV-G Pseudotyped HIV-1 Particles 

HIV-1 particles were obtained from HEK 293 cells co-transfected with a combination of three vectors: psPAX2 pWPXLd-GFP and pHEF-VSV-G. HEK 293 cells were grown in Dulbecco’s modified Eagle medium (DMEM, Sigma, St. Louis, MI, USA) supplemented with 10% fetal bovine serum (Sigma) and 1% L-glutamine (Sigma) at 37 °C under 5% CO_2_. The day before transfection, the cells were plated at a concentration of 3 × 10^5^ cells per well. The following day, the cells were transfected with the appropriate vectors (0.4 mg each) using polyethylenimine (PEI, 1 mg/mL) at a 2:1 PEI:DNA ratio. Four hours after transfection, the culture medium was replaced with fresh DMEM. At 48 h post-transfection, the culture media containing released virions were harvested, filtered through 0.45-µm pore membranes and used for the immunochemical quantification and characterization by ELISA and western blot using rabbit anti-HIV-1 CA antibody.

### 3.7. Enzyme-Linked Immunosorbent Assay (ELISA)

To normalize the amount of VSV-G pseudotyped HIV-1 particles used for the infection of HEK 293 cells, the CA content of the particles was determined by sandwich ELISA. The calibration curve was measured using recombinant purified HIV-1 CA protein [64] in the concentration range of 1.4–8.0 ng/mL in 0.01 M PBS containing 0.05% Tween-20. All samples were analyzed in triplicates in a 96-well plate, which was coated with 100 µL of rabbit anti-HIV-1 CA polyclonal antibody (1:4000 dilution) in 0.05 M carbonate-bicarbonate buffer, pH 9.6, overnight at 4 °C. The plate was then washed three times with 0.01 M PBS containing 0.05% Tween-20 for 10 min at room temperature. The culture medium containing VSV-G pseudotyped HIV-1 particles was filtered through a 0.45 µM filter (Amicon) and diluted (1:100) with 0.01 M PBS. The viral particles were lysed in 0.01 M PBS containing 1% Triton X-100 for 20 min at room temperature. Aliquots (100 µL) of the lysed HIV-1 particles or recombinant HIV-1 CA protein were added to the wells and incubated for 1 h at 37 °C. The plate was then washed three times with 0.01 M PBS containing 0.05% Tween-20 for 10 min at room temperature, and 100 µL of rabbit anti-HIV-1 CA antibody conjugated with horseradish peroxidase (HRP; 1:1,000 dilution in 0.01 M PBS containing 0.05% Tween-20) was added. After 1 h incubation at 37 °C, the plate was washed four times, and 100 µL of the substrate (1 mg of 3,3′,5,5′-tetramethylbenzidine from Sigma-Aldrich diluted in 1 mL.

DMSO, 0.006% hydrogen peroxide, 0.05 M phosphate-citrate buffer, pH 5.0, from Sigma-Aldrich) was added. The reaction was stopped by addition of 50 µL of 2 M sulfuric acid and the absorbance of the samples was measured at 450 nm using a Tecan M200 Pro. The HIV-1 CA amount was calculated using linear regression equation in the linear part of the calibration curve. 

### 3.8. Single-Round Infectivity Assay

Infectivity was determined similarly as described for Mason-Pfizer monkey virus [65,66,67,68,69,70,71]. Briefly, culture media from HEK 293 cells transfected with psPAX2, pWPXLd-GFP, and pHEF-VSV-G vectors in a 1:1:1 ratio in the presence of test compounds were collected 48 h post-transfection and filtered through a 0.45-µm filter. HIV-1 CA content was determined by ELISA. Freshly seeded HEK 293 cells were infected with ELISA-normalized amounts of VSV-G pseudotyped HIV-1 particles, tested PF74 derivatives or DMSO (as a control) were added at various concentrations and incubated for 48 h. The cells were fixed with 2% paraformaldehyde and transferred into a fluorescent activated cell sorting (FACS) tube. Quantification of GFP-positive cells was performed using a BD FACS Aria III flow cytometer.

### 3.9. Flow Cytometry

The sample preparation and measurements were carried out as described elsewhere [68,70,71]. The VSV-G GFP-HIV infected cells were analyzed with a BD FACS Aria III flow cytometer (Becton Dickinson) with the excitation at 488 nm and the emission separated by a 530/30 band pass filter. The obtained data were analyzed with Diva 8 software. In total, 10,000 events were evaluated per sample. First, forward (FSC-A) and side scatter (SSC-A) parameters were used to eliminate dead cells and debris and to yield population P1. The SSC-A and FSC-H parameters were used to analyze only single cells and thus obtain population P2. Using a mock (non-infected, GFP-negative) sample, a proper gating of the GFP-positive cell population was performed for all samples.

### 3.10. Synthesis of PF74 Derivatives 

The PF74 molecule consisted of three synthons: 2-(2-methyl-1H-indol-3-yl)acetic acid, L-phenylalanine and N-methylaniline. All compounds were substituted at the site of the 2-(2-methyl-1H-indol-3-yl)acetamide. Phenylalanine N-methylanilide was connected to the carboxylic group of the synthons (D7,D8 (R),D9) replacing an indole-like structure in the PF74 analogs. Formation of the amide bond was carried out with the aid of the coupling agent T3P^®^. The resulting compounds were purified by column chromatography on silica gel and analytically characterized by NMR and mass spectrometry. The thin-layer chromatography (TLC) was performed using (TLC Silica gel 60 F254, Merck, Darmstadt, Germany). TLC detection (254 nm). The column chromatography was performed using silica gel (100-160 μm, Merck) and a glass column with 2 cm in diameter and 12 cm in height. The NMR spectra were acquired using device from Agilent Technologies with a working frequency of 400 MHz, a chemical shift in ppm (δ), and J-constants in Hz. The orientation LC-MS spectra were acquired using HP series 1100 with quadrupole ionization (Agilent Technologies 6130) with a C_18_ column. The mass spectra of the final compounds were measured using LC-MS TSQ Quantum Access Max (Thermo). The final products were identified via ^1^H NMR, ^13^C NMR, Correlation spectroscopy (COSY), HMQC, and MS.

**Figure 7:**
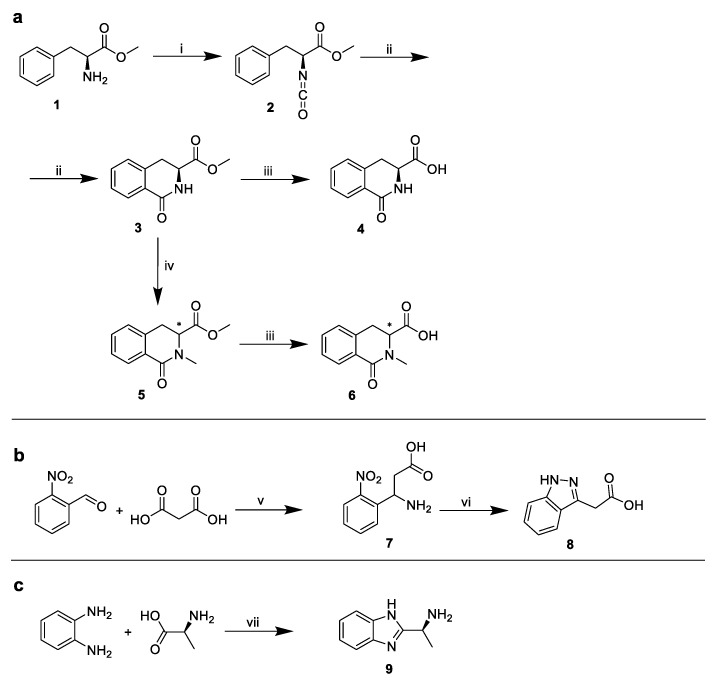
Syntheses of corresponding indole substituents. (**a**) Synthesis of 4 and 6, (**b**) synthesis of 8, (**c**) synthesis of 9. i: triphosgene/NaHCO_3_, ii: AlCl_3_, iii: aq. 5% triethylamine, iv: MeI/NaH, v: ammonium formate, formic acid, vi: Raney nickel, hydrazine hydrate, vii: H_3_PO_4_/polyphosphoric acid.

Due to the commercial inaccessibility of the corresponding indole substituents, the following structures were synthesized (Figure 7):

#### 3.10.1. Methyl (S)-2-isocyanato-3-phenylpropanoate (1)

To a suspension of methyl l-phenylalaninate (700 mg, 3.9 mmol) in CH_2_Cl_2_ (10 mL) at 0 °C we added saturated aqueous NaHCO_3_ (10 mL) and triphosgene (387 mg, 1.3 mmol) in a single portion with vigorous stirring. The reaction mixture was stirred at 0 °C for 1 h and then poured into a separatory funnel. The organic phase was washed with brine, dried over Na_2_SO_4_, filtered, and the solvent was evaporated to give product 1 as a colorless oil (0.6462 g, 81%) which was used in the next step without further purification. ^1^H NMR (CDCl_3_, 400 MHz): δ 7.37–7.24 (m, 3H,), 7.23–7.17 (m, 2H), 4.27 (dd, *J* = 7.8, 4.6 Hz, 1H), 3.81 (s, 3H), 3.16 (dd, *J* = 13.8, 4.6 Hz, 1H), 3.03 (dd, *J* = 13.8, 4.6 Hz, 1H).

#### 3.10.2. Methyl (S)-1-oxo-1,2,3,4-tetrahydroisoquinoline-3-carboxylate (2)

To a solution of compound **1** (0.640 g, 3.11 mmol) in dry CH_2_Cl_2_ (10 mL) AlCl_3_ (0.837 mg, 6.25 mmol) was added and the resulting mixture was refluxed for 3 h. The reaction mixture was cooled to room temperature and then placed in an ice-water bath. Water (8 mL) was slowly added and the mixture was stirred for 30 min. The organic layer was separated and washed with brine. The organic phase was then dried over Na_2_SO_4_. Compound 2 was isolated by evaporation was purified via column chromatography (silica gel, hexane/ethyl acetate (EtOAc), 1/1). Yield 348.8 mg, 55%. TLC (hexane:EtOAc, 1:1 *v*/*v*): Rf = 0.21; ^1^H NMR (CDCl_3_, 400 MHz): δ 7.97 (dd, *J* = 7.7, 1.1 Hz, 1H), 7.38–7.21 (m, 3H), 7.13 (d, *J* = 7.5 Hz, 1H), 4.34 (ddd, = 8.2, 5.5, 2.5 Hz, 1H), 3.64 (s, 3H), 3.25 (dd, *J* = 15.8, 5.5 Hz, 1H), 3.12 dd, *J* = 15.8, 5.5 Hz, 1H); MS: for C_11_H_12_NO_3_ (M+H^+^) *m*/*z* 206.1 found; 206.1 calculated.

#### 3.10.3. Triethylammonium Salt of (S)-1-oxo-1,2,3,4-tetrahydroisoquinoline-3-carboxylic Acid (3)

We dissolved 105 mg (0.51 mmol) of compound **2** in 8 mL of 5% *N*-triethylamine in water and stirred for 2 h. The reaction mixture was freeze dried. Compound 4 was isolated as a triethylammonium salt without any further purification assuming quantitative conversion (0.150 g, ~100%). TLC (CH_2_Cl_2_:MeOH, 5:1 *v*/*v*): Rf = 0.58; ^1^H NMR (CD_3_OD, 400 MHz): δ 7.96 (dd, *J* = 7.7, 1.1 Hz, 1H), 7.35 (td, *J* = 7.5, 1.4 Hz, 1H), 7.28–7.20 (m, 1H), 7.17 (d, *J* = 7.5 Hz, 1H), 6.78 (s, 1H), 4.09 (ddd, *J* = 12.9, 4.4, 1.0 Hz, 1H), 3.30–3.16 (m, 2H), 3.09–2.91 (m, 6H), 1.21 (t, *J* = 7.3 Hz, 9H); MS: for C_10_H_10_NO_3_ (M + H^+^) *m*/*z* 192.1 found; 192.1 calculated.

#### 3.10.4. Methyl 2-methyl-1-oxo-1,2,3,4-tetrahydroisoquinoline-3-carboxylate (4)

NaH (60% dispersion in mineral oil; 56.5 mg, 1.42 mmol) was slowly added to a stirred solution of 2 (116 mg, 0.57 mmol) in DMF (10 mL). MeI (160 mg, 70.4 μL, 1.13 mmol) was added subsequently. The mixture was stirred at 80 °C for 1 h. The reaction was quenched with water (8 mL) at 0 °C and extracted with CH_2_Cl_2_. The combined extracts were washed with water and brine and dried over Na_2_SO_4_. The solvent was removed. The crude product was purified by column chromatography (silica gel, hexane/EtOAc, 1/1) to give compound 4. Yield 82.3 mg, 66%. TLC (hexane:EtOAc, 1:1 *v*/*v*): Rf = 0.33; ^1^H NMR (CDCl_3_, 400 MHz): δ 8.02 (dd, *J* = 7.7 1.1 Hz, 1H), 7.35 (td, *J* = 7.5, 1.4 Hz, 1H), 7.38–7.32 (m, 1H), 7.08 (dd, *J* = 14.9, 5.4 Hz, 1H), 4.21 (dd, *J* = 6.8, 2.0 Hz, 1H), 3.58 (s, 3H), 3.43 (dd, *J* = 16.1,6.8 Hz, 1H), 3.26–3.18 (m, 1H), 3.13 (s, 3H); MS: for C_12_H_14_NO_3_ (M + H^+^) *m*/*z* 220.1 found; 220.1 calculated.

#### 3.10.5. Triethylammonium Salt of 2-methyl-1-oxo-1,2,3,4-tetrahydroisoquinoline-3-carboxylic Acid (5)

We dissolved 82.3 mg (0.38 mmol) of compound **4** in 8 mL of 5% triethylamine in water and stirred for 2 h. The reaction mixture was freeze dried. Compound 5 was isolated as a triethylammonium salt without any further purification assuming quantitative conversion. Yield 113.9 mg, 99%. TLC (CH_2_Cl_2_:MeOH, 5:1 *v*/*v*): Rf = 0.72; ^1^H NMR (CD_3_OD, 400 MHz): δ 7.98 (dd, J = 7.7 1.1 Hz, 1H), 7.32 (td, J = 7.5, 1.4 Hz, 1H), 7.36-7.30 (m, 1H), 7.06 (dd, J = 14.9, 5.4 Hz, 1H), 6.52 (s, 1H), 4.18 (dd, J = 6.8, 2.0 Hz, 1H), 3.42 (dd, J = 16.1,6.8 Hz, 1H), 3.24-3.16 (m, 1H), 3.09 (s, 3H), 3.08–2.92 (m, 6H), 1.24 (t, J = 7.3 Hz, 9H); MS: for C_11_H_12_NO_3_ (M+H^+^) *m/z* 206.8 found; 206.8 calculated.

#### 3.10.6. 3-Amino-3-(2-nitrophenyl)propanoic Acid (6)

2-Nitrobenzaldehyde (2 g, 13.2 mmol), formic acid (85%, 2.5 mL, 37.8 mmol) and malonic acid (1.8 g, 17.3 mmol) were stirred at 45 °C for half an hour. Then, ammonium formate (2.08 g, 33 mmol) was added, the reaction temperature was raised to 70 °C. We stirred the mixture for 1 h, and then stirred at 95 °C for another 4 h. Concentrated HCl was added (8 mL in 5 min) and the mixture was further stirred, maintaining this temperature for 1 h. After mixture cooling, 5 mL of water was added and extracted with EtOAc. The aqueous phase was adjusted to a pH of 4 with 50% NaOH solution. A slightly yellow solid was obtained. The product was dried over NaOH to obtain 662.4 mg of compound 6 (yield 24%). TLC (CH_2_Cl_2_:MeOH, 10:1 *v*/*v*): Rf = 0.35; ^1^H NMR (CDCl_3_, 400 MHz): δ 7.19–7.23 (m, 1H), 6.78–6.68 (m, 3H), 4.41 (q, *J* = 5.4 Hz, 1H), 2.21 (dd, *J* = 12.6, 5.7 Hz, 2H); MS: for C_9_H_11_N_2_O_4_ (M+H^+^) *m*/*z* 210.1 found; 210.1 calculated.

#### 3.10.7. 2-(1. H-indazol-3-yl)acetic Acid (7)

The compound 6 (502 mg, 2.4 mmol) was dissolved in 2.8 mL of aqueous solution of 5% NaOH and then 98% hydrazine hydrate (160 μL) was added. The reaction was heated to 80 °C, and Raney nickel (5 mg) reduction was carried out. After 30 min, the reaction mixture was cooled and adjusted to a pH of 2 with concentrated HCl. The precipitated solid was filtered off and dried over NaOH in desiccator to obtain the compound 8 (329.9 mg, 78%). TLC (CH_2_Cl_2_:MeOH, 3:1 *v*/*v*): Rf = 0.53; ^1^H NMR (CD_3_OD, 400 MHz): δ 7.76–7.71 (m, 1H), 7.47 (d, *J* = 8.5 Hz, 1H), 7.39-7.33 (m, 1H), 7.12 (t, *J* = 7.5 1H), 3.98 (s, 2H); MS: for C_9_H_9_N_2_O_2_ (M + H^+^) *m*/*z* 176.1 found; 176.1 calculated.

#### 3.10.8. (S)-1-(1H-benzo[d]imidazol-2-yl)ethan-1-amine (8)

*o*-Phenylenediamine (50 mg, 0.46 mmol), l-alanine (42 mg, 0.47 mmol), polyphosphoric acid (10 mg), and H_3_PO_4_ (300 μL, 5.6 mmol) were heated at 150 °C in the microwave reactor for 2 h (normal absorption mode). The mixture was diluted with 500 μL of water and the pH was adjusted to approximately 9 by a saturated aqueous solution of NaOH. The reaction mixture was diluted with 5 mL of EtOH, cooled to room temperature, and filtered. The solvent from the filtrate was evaporated and the crude product was purified with the aid of column chromatography (silica gel, DCM/MeOH, 5:1,). 33.2 mg of compound 8 with 45% yield was obtained. TLC (CH_2_Cl_2_:MeOH, 5:1 *v*/*v*): Rf = 0.34; ^1^H NMR (CD_3_OD, 400 MHz): δ 11.82 (s, 1H), 7.49–7.45 (m, 2H), 7.12-7.08 (m, 2H), 4.35 (dd, *J* = 11.2, 8.4 Hz, 1H), 3.25 (s, 1H), 1.89 (d, *J* = 13.2 Hz, 3H); MS: for C_9_H_12_N_3_ (M + H^+^) *m*/*z* 162.2 found; 162.2 calculated.

**Figure 8:**
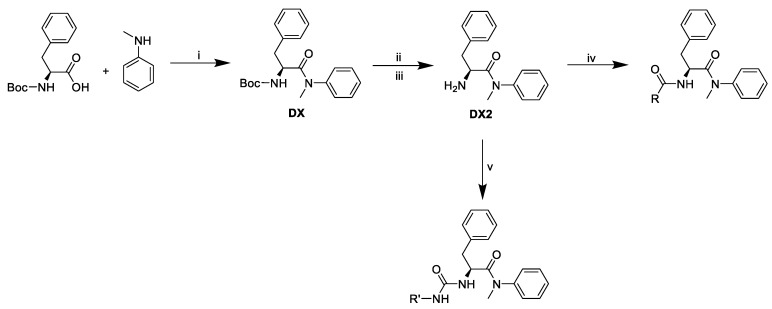
Synthetic pathway of final derivatives. i: T3P^®^, pyridine, ii: trifluoroacetic acid, iii: Dowex^®^ 1 × 8 in OH^-^ form, iv: T3P^®^, pyridine, R-COOH, v: triphosgene, *N*,*N*-diisopropylethylamine, R-NH_2_.

Pathway to final compounds (Figure 8).

#### 3.10.9. Tert-butyl (S)-(1-(methyl(phenyl)amino)-1-oxo-3-phenylpropan-2-yl)carbamate (DX).

Boc-l-phenylalanine (100 mg, 0.38 mmol) was dissolved in a solution of pyridine and EtOAc (5 mL, 3:1, *v*/*v*). *N*-methylaniline (61.3 μL, 0.57 mmol) and T3P^®^ (449 μL, 0.75 mmol) were added. The reaction was stirred for 5 h or overnight. The reaction mixture was diluted with EtOAc (5 ml) and transferred to the separation funnel. The extraction was carried out with 0.5 M HCl and with 0.5 M NaHCO_3_. The organic phase was dried over dry Na_2_SO_4_ and evaporated. The crude product was then purified using column chromatography (silica gel, hexane/EtOAc, 2:1). 104.2 mg, 77% yield. TLC (hexane:EtOAc, 5:1 *v*/*v*): Rf = 0.37; ^1^H NMR (CDCl_3_, 400 MHz): δ 7.34-7.14 (m, 2H), 7.15–6.99 (m, 3H), 6.92-6.62 (m, 5H), 5.47 (d, *J* = 8.8 Hz, 1H), 4.46 (dd, *J* = 15.1, 7.2 Hz, 1H), 3.10 (s, 3H), 2.87–2.55 (m, 1H), 1.26 (s, 9H); MS: for C_21_H_27_N_2_O_3_ (M + H^+^) *m*/*z* 355.4 found; 355.4 calculated.

#### 3.10.10. (S)-2-amino-N-methyl-N,3-diphenylpropanamide (DX2)

DX (100 mg, 0.28 mmol) was dissolved in 2.5 mL of CH_2_Cl_2_ and 2.5 mL of trifluoroacetic acid was added while stirred. Trifluoroacetic acid was evaporated and after 30 min, the product was dissolved in CH_2_Cl_2_ and subsequently stirred with preactivated Dowex^®^ 1 × 8 in hydroxide form to remove trifluoroacetic anion. The obtained product was used directly for the next reaction step. TLC (CH_2_Cl_2_:MeOH, 100:1 *v*/*v*): Rf = 0.19; MS: for C_16_H_19_N_2_O (M + H^+^) *m*/*z* 255.1 found; 255.1 calculated.

#### 3.10.11. (S)-2-(2-(1H-indol-3-yl)acetamido)-N-methyl-N,3-diphenylpropanamide (D1)

The condensations of compound DX2 (35.9 mg, 0.14 mmol) and 2-(1*H*-indol-3-yl)acetic acid (16.4 mg, 0.09 mmol) were carried out according to the general method (pg. 13). The compound was purified using column chromatography (silica gel, hexane/EtOAc, 1:1). It was obtained at 34.7 mg, yield 60%. TLC (hexane:EtOAc, 1:1 *v*/*v*): Rf = 0.17; ^1^H NMR (CDCl_3_, 400 MHz): δ 8.44 (s, 1H), 7.45 (d, *J* = 7.9 Hz, 1H), 7.34 (ddd, 6.75, 5.9, 5.0 Hz, 4H), 7.24–7.18 (m, 1H), 7.16–7.14 (m, 1H), 7.13 (d, *J* = 1.8 Hz, 1H), 7.11 (d, *J* = 3.1 Hz, 2H), 7.08 (s, 1H), 7.06 (s, 1H), 6.98 (d, *J* = 2.2 Hz, 1H), 6.70 (d, *J* = 7.1 Hz, 2H), 6.30 (d, *J* = 8.3 Hz, 1H), 4.82 (dd, *J* = 15.3, 7.1 Hz, 1H), 3.66 (d, *J* = 1.7 Hz, 2H), 3.17 (s, 3H), 2.76 (dd, *J* = 13.3, 7.1 Hz, 1H), 2.57 (dd, *J* = 13.3, 7.1 Hz, 1H); ^13^C NMR (CDCl_3_, 400 MHz): δ 171.36, 170.76, 142.40, 136.35, 136.05, 129.73, 129.17, 128.26, 128.11, 127.29, 127.04, 126.68, 123.67, 122.35, 119.82, 118.68, 111.32, 108.64, 51.10, 38.81, 37.60, 33.28; HRMS/ESI: for C_26_H_26_N_3_O_2_ (M + H^+^) *m*/*z* 412.20226 found; 412.20195 calculated, for C_26_H_25_N_3_O_2_Na (M + Na^+^) *m*/*z* 434.18420 found; 434.18390 calculated.

#### 3.10.12. (S)-2-(2-(1-(4-chlorobenzoyl)-5-methoxy-2-methyl-1H-indol-3-yl)acetamido)-N-methyl--N,3-diphenylpropanamide (D2)

The condensations of compound DX2 (46.6 mg, 0.18 mmol) and indomethacin (43.6 mg, 0.12 mmol) were carried out according to general method. The compound was purified using column chromatography (silica gel, hexane/EtOAc, 2:1). It was obtained at 62.9 mg, yield 58%. TLC (hexane:EtOAc, 1:1 *v*/*v*): Rf = 0.47; ^1^H NMR (CDCl_3_, 400 MHz): δ 7.66–7.62 (m, 2H), 7.50–7.45 (m, 2H), 7.41–7.32 (m, 3H), 7.12 (ddd, *J* = 6.3, 3.7, 1.3 Hz, 1H), 7.09–7.03 (m, 2H), 7.01 (s, 1H), 6.98 (d, *J* = 6.3 Hz, 2H), 6.84 (d, J = 2.5 Hz, 1H), 7.66–7.62 (m, 1H), 6.70–6.66 (m, 2H), 6.25 (d, *J* = 8.1 Hz, 1H), 4.81 (dd, *J* = 14.9, 6.9 Hz, 1H), 3.80 (s, 3H), 3.55 (d, *J* = 5.1 Hz, 2H), 3.21 (s, 3H), 2.78 (dd, *J* = 13.4, 6.6 Hz, 1H), 2.58 (dd, *J* = 13.4, 7.0 Hz, 1H), 2.4 (s, 3H); ^13^C NMR (CDCl_3_, 400 MHz): δ 171.02, 169.13, 168.27, 156.24, 142.42, 139.26, 135.96, 135.86, 133.80, 131.22, 130.96, 130.30, 129.92, 129.15, 129.10, 128.27, 128.18, 127.31, 126.75, 115.10, 112.81, 112.31, 100.85, 55.72, 51.15, 38.52, 37.67, 32.11, 13.41; HRMS/ESI: for C_35_H_33_ClN_3_O_4_ (M+H^+^) *m*/*z* 594.21566 found; 594.21541 calculated, for C_35_H_32_ClN_3_O_4_Na (M + Na^+^) *m*/*z* 616.19782 found; 616.19736 calculated, for C_35_H_32_ClN_3_O_4_K (M + K^+^) *m*/*z* 632.17092 found; 632.17129 calculated.

#### 3.10.13. (S)-2-((R)-2-(4-isobutylphenyl)propanamido)-N-methyl-N,3-diphenylpropanamide (D4(R))

The condensations of compound **DX2** (57.4 mg, 0.23 mmol) and racemic ibuprofen (31 mg, 0.15 mmol) were carried out according to the general method. The compound was purified using column chromatography (silica gel, hexane/EtOAc, 5:1). The yields of both diastereomers were 68% (67.6 mg). The yield of D4(*R*) was 30% (29.8 mg). The product was identified by comparison with an optically pure standard prepared in the same procedure using the *R*-ibuprofen. TLC (hexane:EtOAc, 3:1 *v*/*v*): Rf = 0.15; ^1^H NMR (CDCl_3_, 400 MHz): δ 7.35 (tdd, *J* = 7.45, 5.9, 1.7 Hz, 2H), 7.18–7.12 (m, 1H), 7.09 (t, *J* = 7.3 Hz, 3H), 7.05 (s, 4H), 6.97 (d, *J* = 6.3 Hz, 2H), 6.67 (d, *J* = 7.1 Hz, 2H), 6.03 (s, 1H), 4.84 (dd, *J* = 15.3, 7.0 Hz, 1H), 3.48 (q, *J* = 7.1 Hz, 1H), 3.21 (s, 3H), 2.74 (dd, *J* = 13.4, 6.6 Hz, 1H), 2.54 (dd, *J* = 13.4, 6.6 Hz, 1H), 2.45 (d, *J* = 7.2 Hz, 2H), 1.85 (tt, *J* = 13.5, 6.7 Hz, 1H), 1.40 (d, *J* = 7.1 Hz, 3H), 0.90 (d, *J* = 6.6 Hz, 6H); ^13^C NMR (CDCl_3_, 400 MHz): δ 173.41, 171.44, 142.44, 140.44, 138.48, 136.08, 129.80, 129.48, 129.16, 128.23, 128.11, 127.29, 126.62, 50.91, 46.43, 45.04, 38.70, 37.62, 30.17, 22.39, 18.34; HRMS/ESI: for C_29_H_35_N_2_O_2_ (M + H^+^) *m*/*z* 443.26922 found; 443.26930 calculated, for C_29_H_34_N_2_O_2_Na (M + Na^+^) *m*/*z* 465.25132 found; 465.25125 calculated, for C_29_H_34_N_2_O_2_K (M + K^+^) *m*/*z* 481.22491 found; 481.22519 calculated.

#### 3.10.14. (S)-N-((S)-1-(methyl(phenyl)amino)-1-oxo-3-phenylpropan-2-yl)-1-oxo-1,2,3,4--tetrahydroisoquinoline-3-carboxamide (D7)

The condensations of compound DX2 (35.9 mg, 0.14 mmol) and compound 3 (17.9 mg, 0.09 mmol) were carried out according to the general method. Compound was purified using column chromatography (silica gel, hexane/EtOAc, 3:2). It was obtained 56.8 mg, yield 90%. TLC (EtOAc): Rf = 0.26; ^1^H NMR (CDCl_3_, 400 MHz): δ 8.04 (dd, *J* = 7.6, 1.1 Hz, 1H), 7.69 (d, *J* = 8.3 Hz, 1H), 7.61 (s, 1H), 7.43–7.38 (m, 1H), 7.15–7.11 (m, 1H), 7.10–7.08 (m, 2H), 7.06–7.04 (m, 1H), 7.00–6.93 (m, 2H), 6.73–6.68 (m, 2H), 4.79 (dd, *J* = 14.7, 8.0 Hz, 1H), 4.29 (td, *J* = 6.2, 3.6 Hz, 1H), 3.26 (t, *J* = 6.0 Hz, 2H), 3.20 (s, 3H), 2.83 (dd, *J* = 13.4, 6.4 1H), 2.68 (dd, *J* = 13.4, 6.4 Hz, 1H); ^13^C NMR (CDCl_3_, 400 MHz): δ 171.68, 170.24, 165.52, 142.25, 136.57, 136.12, 132.50, 129.82, 129.02, 128.33, 128.03, 127.52, 127.28, 126.65, 125.94, 124.32, 54.06, 51.75, 38.46, 37.80, 30.94; HRMS/ESI: for C_26_H_25_N_3_O_3_Na (M + Na^+^) *m*/*z* 450.17911 found; 450.17881 calculated, for C_26_H_25_N_3_O_3_K (M + K^+^) *m*/*z* 466.15254 found; 466.15275 calculated.

#### 3.10.15. (R)-2-methyl-N-((S)-1-(methyl(phenyl)amino)-1-oxo-3-phenylpropan-2-yl)-1-oxo--1,2,3,4-tetrahydroisoquinoline-3-carboxamide (D8(R))

The condensations of compound DX2 (39.5 mg, 0.15 mmol) and the compound 5 (21.2 mg, 0.10 mmol) were carried out according to general method. The compound was purified using column chromatography (silica gel, hexane/EtOAc, 3:2). Both obtained diastereomers were further separated. The yields of both diastereomers were 60% (43.2 mg). Yield of D8(*R*) 35% (25.2 mg). TLC (hexane:EtOAc, 3:2 *v*/*v*): Rf = 0.38; ^1^H NMR (CDCl_3_, 400 MHz): δ 8.04 (dd, *J* = 7.5, 1.4 Hz, 1H), 7.39–7.28 (m, 5H, H-8, H10), 7.09–7.01 (m, 3H), 7.00-6.90 (m, 4H), 6.45 (d, *J* = 7.2 Hz, 2H), 4.72 (td, *J* = 8.2, 5.9 Hz, 1H), 4.07 (dd, *J* = 7.1, 2.2 Hz, 1H), 3.35 (dd, *J* = 16.2, 7.2 Hz, 1H), 3.23 (s, 3H), 3.20 (s, 3H), 2.63 (dd, *J* = 13.6, 5.8 Hz, 1H), 2.37 (dd, *J* = 13.6, 5.8 Hz, 1H); ^13^C NMR (CDCl_3_, 400 MHz): δ 170.95, 169.50, 164.29, 142.24, 135.64, 135.15, 132.05, 129.87, 128.68, 128.32, 128.25, 127.97, 127.33, 127.26, 126.60, 61.98, 51.27, 38.47, 37.70, 35.12, 30.67; HRMS/ESI: for C_27_H_27_N_3_O_3_Na (M + Na^+^) *m*/*z* 464.19472 found; 464.19446 calculated, for C_27_H_27_N_3_O_3_K (M + K^+^) *m*/*z* 480.16794 found; 480.16840 calculated.

#### 3.10.16. (S)-2-methyl-N-((S)-1-(methyl(phenyl)amino)-1-oxo-3-phenylpropan-2-yl)-1-oxo--1,2,3,4-tetrahydroisoquinoline-3-carboxamide (D8(S))

The condensations of compound DX2 (39.5 mg, 0.15 mmol) and the compound 5 (21.2 mg, 0.10 mmol) were carried out according to the general method. The compound was purified using column chromatography (silica gel, hexane/EtOAc, 3:2). Both obtained diastereomers were further separated. The yields of both diastereomers were 60% (43.2 mg). The yield of D8(*S*) was 25% (18 mg). TLC (hexane:EtOAc, 3:2 *v/v)*: Rf = 0.22; ^1^H NMR (CDCl_3_, 400 MHz): δ 8.07 (dd, *J* = 7.6, 1.3 Hz, 1H), 7.39 (tt, *J* = 6.1, 3.0 Hz, 1H), 7.36–7.29 (m, 1H), 7.29–7.24 (m, 3H), 7.21–7.14 (m, 3H), 7.08 (d, *J* = 7.4 Hz, 1H), 6.82 (dd, *J* = 7.1, 2.3 Hz, 2H), 6.71–6.65 (m, 2H), 6.60–6.53 (m, 1H), 4.65 (dd, *J* = 15.3, 7.3 Hz, 1H), 4.05 (dd, *J* = 6.8, 2.3 Hz, 1H), 3.14 (dd, *J* = 16.1, 2.5 Hz, 1H), 3.10 (s, 3H), 3.07 (s, 3H), 2.84 (dd, *J* = 13.3, 7.2 Hz, 1H), 2.61 (dd, *J* = 13.3, 7.2 Hz, 1H); ^13^C NMR (CDCl_3_, 400 MHz): δ 170.70, 169.16, 164.53, 142.15, 135.98, 134.95, 131.86, 129.77, 129.17, 128.40, 128.17, 128.11, 127.40, 127.05, 126.86, 61.81, 51.23, 38.60, 37.56, 34.94, 31.16; HRMS/ESI: for C_27_H_27_N_3_O_3_Na (M + Na^+^) *m*/*z* 464.19473 found; 464.19446 calculated, for C_27_H_27_N_3_O_3_K (M + K^+^) *m*/*z* 480.16840 found; 480.16840 calculated.

#### 3.10.17. (S)-2-(2-(1H-indazol-3-yl)acetamido)-N-methyl-N,3-diphenylpropanamide (D9)

The condensations of compound DX2 (37.3 mg, 0.15 mmol) and compound 7 (17.1 mg, 0.10 mmol) were carried out according to the general method. The compound was purified using column chromatography (silica gel, hexane/EtOAc, 2:1). It was obtained at 36.2 mg, yield 57%. TLC (hexane:EtOAc, 2:1 *v*/*v*): Rf = 0.23; ^1^H NMR (CDCl_3_, 400 MHz): δ 8.13 (d, *J* = 8.5 Hz, 1H), 7.73 (d, *J* = 8.1 Hz, 1H), 7.43 (d, *J* = 8.4 Hz, 1H), 7.34 (dd, *J* = 8.0, 7.2 Hz, 1H), 7.30–7.24 (m, 3H), 7.15–7.01 (m, 4H), 6.85-6.73 (m, 4H), 4.88 (dd, *J* = 15.2, 8.0 Hz, 1H), 4.09-3.91 (m, 2H), 3.21 (s, 3H), 2.90 (dd, *J* = 13.2, 8.0 Hz, 1H), 2.70 (dd, *J* = 13.2, 8.0 Hz, 1H); ^13^C NMR (CDCl_3_, 400 MHz): δ 172.00, 168.90, 142.21, 141.00, 140.27, 136.20, 129.61, 129.25, 128.21, 128.07, 127.25, 126.71, 126.66, 122.18, 120.55, 120.38, 110.18, 51.39, 39.15, 37.73, 35.35; HRMS/ES: for C_25_H_24_N_4_O_2_Na (M + Na^+^) *m*/*z* 435.17920 found; 435.17915 calculated, for C_27_H_27_N_3_O_3_K (M + K^+^) *m*/*z* 451.15289 found; 451.15308 calculated.

#### 3.10.18. (S)-2-(3-((1H-benzo[d]imidazol-2-yl)methyl)ureido)-N-methyl-N,3-diphenylpropanamide (D10)(General procedure of urea-linked derivatives preparation)

The compound DX2 (45.4 mg, 0.18 mmol) was dissolved in 5 mL of CH_2_Cl_2_ and cooled to 0 °C. 

*N*,*N*-diisopropylethylamine (311 μL, 1.78 mmol) and triphosgene (17.5 mg, 0.06 mmol) were then added. The (1*H*-benzo[*d*]imidazol-2-yl)methanamine dihydrochloride (59.4 mg, 0.27 mmol) was added after 30 min. The mixture was stirred at room temperature overnight. The solvent was evaporated and obtained compound D10 was crystallized from acetonitrile. We obtained 21.6 mg, yield 28%. TLC (CH_2_Cl_2_:MeOH, 10:1 *v*/*v*): Rf = 0.59; ^1^H NMR (CD_3_OD, 600 MHz): δ 7.53 (dd, *J* = 5.8, 3.2 Hz, 2H), 7.43–7.36 (m, 2H, H-8), 7.22 (dd, *J* = 6.0, 3.2 Hz, 2H), 7.20–7.16 (m, 3H), 7.09 (s, 2H), 6.88 (dd, *J* = 6.4, 2.7 Hz, 2H), 4.87 (s, 2H), 4.62–4.55 (m, 2H), 4.50 (d, *J* = 16.4 Hz, 1H), 3.24 (s, 3H), 2.94 (dd, *J* = 13.4, 6.5 Hz, 1H), 2.70 (dd, *J* = 13.4, 8.1 Hz, 1H); ^13^C NMR (CD_3_OD, 600 MHz): δ 171.30, 157.82, 153.43, 143.40, 137.95, 129.96, 129.35, 128.56, 128.19, 127.96, 126.81, 122.32, 118.96, 115.93, 52.40, 38.68, 38.13, 37.58; HRMS/ESI: for C_25_H_26_N_5_O_2_ (M + H^+^) *m*/*z* 428.20815 found; 428.20810 calculated, for C_25_H_25_N_5_O_2_Na (M + Na^+^) *m*/*z* 450.19001 found; 450.19005 calculated, for C_25_H_25_N_5_O_2_K (M + K^+^) *m*/*z* 466.16356 found; 466.16398 calculated.

#### 3.10.19. (S)-2-(3-((S)-1-(1H-benzo[d]imidazol-2-yl)ethyl)ureido)-N-methyl-N,3--diphenylpropanamide (D11)

The connection of DX2 (46.6 mg, 0.18 mmol) to the compound 8 (43.5 mg, 0.27 mmol) was done through a urea bridge according to the general procedure (see above). It was purified using column chromatography (silica gel, DCM/MeOH, 30:1). We obtained 25 mg, yield 33%. TLC (CH_2_Cl_2_:MeOH, 10:1 *v*/*v*): Rf = 0.63; ^1^H NMR (DMSO-*d_6_*, 600 MHz): δ 7.68 (s, 2H), 7.46-7.32 (m, 3H), 7.18 (d, *J* = 6.6 Hz, 2H), 7.13-7.03 (m, 3H), 6.83 (d, *J* = 5.0 Hz, 2H), 6.76 (d, *J* = 7.0 Hz, 2H), 6.61 (d, *J* = 8.2 Hz, 1H), 5.05–4.98 (m, 1H), 4.18–4.13 (m, 1H), 3.52 (s, 2H), 3.15 (s, 3H), 2.79 (dd, *J* = 13.4, 4.5 Hz, 1H), 2.53 (dd, *J* = 13.4, 4.5 Hz, 1H), 1.54 (d, *J* = 6.8 Hz, 3H); ^13^C NMR (DSMO-*d_6_*, 600 MHz): δ 172.14, 157.32, 157.01, 143.30, 141.23, 137.76, 130.00, 129.31, 128.52, 128.24, 127.92, 126.77, 124.95, 114.71, 52.33, 43.93, 38.63, 37.58, 19.38; HRMS/ESI: for C_26_H_28_N_5_O_2_ (M + H^+^) *m*/*z* 422.22388 found; 422.22375 calculated, for C_26_H_27_N_5_O_2_Na (M + Na^+^) *m*/*z* 464.20559 found; 464.20570 calculated, for C_26_H_27_N_5_O_2_K (M + K^+^) *m*/*z* 480.17923 found; 480.17963 calculated.

### 3.11. The Cyclosporin A (CsA) Washout Assay

Owl monkey kidney (OMK) cells were seeded in a 48-well plate at a concentration of 15,000 cells per well. The next day, Eagle’s Minimum Essential Medium (EMEM) was replaced with medium containing 2.5 µM CsA and polybrene (5 ng/µL), and the OMK cells were spinoculated for 1 h with a normalized amount of HIV-1 particles in the presence or absence of PF74 and D7, D8(R), and D9 derivatives (10 µM). Two hours after infection, the medium was replaced with fresh medium containing 2.5 µM CsA. At various times, the cultivation medium containing CsA was removed and replaced with fresh medium without CsA. After 48 h, the cells were fixed with 2% formaldehyde and GFP-positive cells were counted using flow cytometry (BD FACS AriaIII).

### 3.12. NMR Titration

The NMR sample contained 0.6 mM CA-NTD in phosphate buffer, 100 mM NaCl (pH 6.0), and 10% D_2_O. The NMR spectra were acquired at 25 deg on Bruker Avance III 600 MHz equipped with ^15^N/^13^C/^1^H triple resonance, cryogenically cooled probe, and were analyzed in Sparky. The inhibitors were added in DMSO and the ratios were (inhibitor:protein) 1:8, 1:4, 1:2, 1:1, and 2:1. The NMR data were processed in Topspin 3.6 and analyzed in Sparky.

## Figures and Tables

**Table 1:** Cytotoxicity and inhibitory effect of PF74 and its derivatives.

Compound	PF74	D1	D2	D4R	D7	D8S	D8R	D9	D10	D11
**CC_50_ (µM)**	>40	>40	>40	27 ± 3.5	>40	>40	>40	>40	10 ± 1.6	>40
**IC_50_ (µM)**	1.75 ± 0.5	3.5 ± 0.6	8.0 ± 1.2	>20	>20	>20	4.5 ± 1.5	3.4 ± 0.9	0.5 ± 0.3	15 ± 3
